# Diseases of the Surrounding Tissues Originating in Carious Teeth

**Published:** 1854-01

**Authors:** C. Bate Spence

**Affiliations:** 8 Mulgrave Place, Plymouth, England.


### ARTICLE XV.

*Diseases of the Surrounding Tissues Originating in Carious Teeth.* By C. Bate Spence, Esq., 8 Mulgrave Place, Plymouth, England.

I have said, that one of the greatest evils which can result from an irregularity of the teeth is the predisposition which they have to be affected by caries on the surfaces which approximate each other, not that their pressing one against the other inflicts any injury on the structure of the dentinal tissues, but that their close approximation offers convenient positions for the perpetual lodgment of the juices of the mouth, which, although not injurious in themselves, yet, being the vehicle which holds in solution the elements which chemically carry away the salts of the teeth, those parts of the teeth must be much more liable to be acted upon which are so placed as to be mostly under their influence; in other words, those spots where saliva always rests.

Nor will the evil stay itself in the loss of the teeth; caries of the tooth produces exposure of the pulp to many abnormal influences, under which disease and pain are produced. This terminates sometimes in the forcible extraction of the tooth affected, sometimes in more serious ills, which latter I have generally found to be the more important where the tooth has been destroyed under the ravages of caries, without inducing inflammatory action of the pulp, and, consequently, without producing pain, which circumstance, perhaps, may in itself be a leading, though indirect, cause of the collateral malady, an example of which may be seen in the following case of Mrs. T. A. M., a married lady of somewhat about forty-five years of age, the mother of a family, all of them healthy.

It was while extracting a tooth at the house of one of my patients, that the lady to whom this present case refers requested me to observe a swelling of which she had been conscious for some little while to exist at the side of her nose beneath the left eye. It was not very large, but painless, and exceedingly hard. Upon examining the state of the internal mouth, my attention was drawn by herself to a decayed upper first molar upon the same side. It had never pained, was firmly fixed, and showed no symptoms of alveolar disturbance; the remainder of the teeth upon that side were generally sound and healthy; and I thought myself justified in confirming her notion in attributing the possibility of the tumor to have originated from this source, even though it existed at some distance from the irritating cause.

Having taken this view of the case, on which diagnosis an advantage existed in being on the safe side, since the treatment which should follow could only affect a diseased and almost useless organ, the removal of the decayed tooth I suggested as being advisable, which gained a ready assent, with the request, that I should operate immediately—a circumstance for which I was not prepared, the forceps which I had with me being for other teeth; and we parted with the understanding that she was to call at my house on the succeeding day.

Instead of fulfilling her portion of the arrangement, a whole year was allowed to pass away before I again saw her, when she sent for me to call upon her, which I did at her own house. Upon seeing her I could scarcely suppress an expression of astonishment at the alteration and great disfigurement which the countenance had undergone. The swelling had enlarged considerably, pressing the nose aside, drawing the corner of the mouth awry, and giving a disagreeable distortion to the whole countenance. A wide separation had also taken place between the left centre and lateral incisors.

The history of the case for the last twelve months was, that in consequence of my not operating immediately, and requesting her to call upon me to have the tooth extractcd the next day, induced in her mind the idea that I must have thought the tooth difficult to extract, and, therefore, created a fear as to the result which she had not previously entertained, but, which having once been aroused, she sought the advice of her usual medical attendant as to the propriety of her losing this carious but painless tooth.

From some inexplicable cause, he advised her by no means to part with it, since there was no evidence to show that the tumor situated in the anterior region of the face was caused by a tooth, though carious, yet situated among the most posterior in the mouth. But twelve months had passed away, and there was no occasion now for any persuasion in order to win her consent to the removal of the molar, to which allusion has already been made as being decayed. I also took advantage of my present influence to induce her to have removed from her mouth every particle of substance which might, under unfavorable circumstances, become sources of irritation to the surrounding soft tissues; for instance, a few fangs of decayed teeth upon the opposite side of the mouth, together with not a little quantity of salivary calculus.

About a week after this period, the tumor, which had previously always been very hard and free from pain, became soft, tender, and even painful, after the manipulation necessary to diagnose its character.

A few days after, it appeared under the finger to fluctuate as if it were filled with some fluid, and showed a disposition to become inflamed upon the external surface, indicating what appeared to me signs of approaching suppuration.

Hailing this as a most fortunate index of the result which might be expected, and wishing to preclude the liability of its burrowing through the cheek, and forming an ugly scar upon the face, I inserted a lancet into the tumor, between the upper lip and the gum; but, instead of a copious flow of pus, as I had hoped, a small quantity only escaped, together with a watery fluid, which latter continued to flow through the opening for some time, the orifice made by the lancet being kept open. Shortly after this, the tumor became painful, although it decreased in size and became soft.

I have previously observed, that a separation had taken place between the lateral and central incisors upon the same side of the mouth. The former of these two teeth, together with the adjoining canine, seemed to yield from their place, as if their original strength of position had become weakened. This not being continued to the central incisors, seemed to point to the separation which was taking place between the lateral and central as being the line of demarcation between the diseased and healthy tissues. The bone posterior to the central incisor was evidently separating from the rest of the bony structure by necrosis. I felt that the proper mode of treatment would be to remove these two otherwise sound teeth, to which a ready consent was given. But although I felt convinced of the correctness of my own opinion, yet the part which I take in the adjustment of artificial teeth induced me to request that a second opinion might be taken, in order to convince herself and family that she would not unnecessarily lose teeth which in themselves were not diseased.

It was some time after this before I saw her again, and which was only in consequence of an express request, when I was alarmed to observe, that the whole system felt the shock to such an extent that health was seriously yielding under it.

I was then requested to carry out my own treatment, in removing the teeth, which I had previously proposed, and do whatever I thought proper; but since my opinion had not been supported by the advice called in during the respite of my visits, I declined to operate, when they said that they should seek assistance in some other neighborhood; this, in consequence of her fast declining strength, I urged upon them to carry out immediately, and which was obtained from the skillful hands of the younger Mr. Travers, who, through the assistance of Professor Bell, instantly removed the teeth in question, a few portions of the alveolar ridge came off by necrosis, after which the tumor disappeared, the opening in the mouth healed up, and the general health rapidly and successfully progressed; but the system was a long time before it quite recovered, even if it ever has completely, from the shock given to it by this local irritation, originating in a decayed but painless tooth.

